# Voice alterations in patients with Morquio A syndrome

**DOI:** 10.1007/s13353-017-0421-6

**Published:** 2017-12-23

**Authors:** Krzysztof Szklanny, Ryszard Gubrynowicz, Anna Tylki-Szymańska

**Affiliations:** 1 Multimedia Department, Polish-Japanese Academy of Information Technology, Warsaw, Poland; 2 Department of Pediatrics, Nutrition and Metabolic Diseases, The Children’s Memorial Health Institute, Warsaw, Poland

**Keywords:** Morquio a syndrome, Metabolic disorders, Genetic disorder, electroglottography, Vocal folds, Voice and speech disorder, voice quality

## Abstract

Morquio A syndrome, or mucopolysaccharidosis (MPS IV A), is an inherited lysosomal storage disorder which belongs to the group of mucopolysaccharidoses (MPSs). It is caused by N-acetylgalactosamine-6-sulfatase (GALNS) activity deficiency, which results in impaired degradation of glycosaminoglycans (GAGs), including keratan sulfate (KS) and chondroitin-6-sulfate (CS). These compounds infiltrate and disrupt the architecture of the extracellular matrix, compromising the integrity of the connective tissue. Patients with Morquio A have also been noted for exhibiting abnormalities of the larynx and vocal tract. The aim of the study was to assess voice alterations using noninvasive acoustic and electroglottographic voice analysis. Electroglottographic signal and acoustic analyses revealed considerable changes in the voices of patients with Morquio A syndrome when compared to the voices of healthy controls. Affected patients tended toward tense voice, incomplete glottal closure, increased incidence of vocal fold nodules, dysphonia, and hoarse voice. Morquio A syndrome is characterized by connective tissue disease, which adversely affects voice quality. The use of objective voice analysis makes it possible to quantitatively monitor changes in the vocal apparatus over the course of disease progression, and also allows for assessment of the effects of the enzyme replacement therapy.

## Introduction

Morquio A syndrome, mucopolysaccharidosis (MPS) IV A, is an autosomal recessive lysosomal storage disorder caused by N-acetylgalactosamine-6-sulfatase (GALNS) activity deficiency. This deficiency leads to the accumulation of glycosaminoglycans (GAGs), including keratan sulfate (KS) and chondroitin-6-sulfate (CS), which in turn infiltrate the connective tissue causing weakness, and disrupt the structure of the extracellular matrix. Patients with Morquio A syndrome are characterized by skeletal and joint abnormalities (joint hypermobility, dwarfism, short trunk, cervical spine abnormalities, and pectus carinatum) (Hendriksz et al. 2014; Hendriksz et al. 2013).

Respiratory and voice manifestations are a consequence of GAG deposits in the upper airway. Upper airway obstruction and obstructive sleep apnea, combined with thoracic cage deformity and tracheal distortion resulting from shortened spinal height, often lead to total airway collapse (Walker 2003; Pelley et al. 2007). Taken together, these abnormalities result in both respiratory problems (Harmatz et al. 2013; Nashed et al. 2009; Yeung et al. 2009) and voice and speech disorders (Hendriksz et al. 2014).

Morquio A syndrome patients are commonly reported to have abnormalities of the larynx (notably a narrowed laryngeal airway due to tissue laryngeal thickening) (Walker 2003) and vocal tract (Harmatz et al. 2015). Deformities are often observed in the laryngeal vestibule, ventricular and true vocal folds, and the mucosa of the posterior region of the larynx, which are all crucial for voice and speech production (Harmatz et al. 2015; Nakarat et al. 2013). Irregularly shaped trachea and/or vocal folds and airway collapse resulting in tracheal and lower-airway obstruction are consequences of the cartilage malformation and mucopolysaccharide deposition in this disease (Pelley et al. 2007).

Acoustic analysis allows for the assessment of voice quality and speech pathology. Importantly, this permits monitoring of changes in the declining function of the speech apparatus over the course of the disease and the potential effects of enzyme replacement therapy. The advantage of this method lies in its noninvasive and repeatable character. So far, acoustic analysis has proven useful in monitoring voice alterations in patients with MPS I, II, and VI, with the aid of the Göttingen hoarseness diagram (Nakarat et al. 2013). Irregularities in the radiated voice signal are thought to be caused by perturbations in vocal fold vibration. These can be characterized as jitter (irregularity in periodicity) and shimmer (irregularity in amplitude). The amount of noise in the radiated signal, which is measured in the glottal to noise excitation ratio, is further increased by turbulent airflow resulting from incomplete glottal closure.

The aim of this study was to describe voice alterations in patients with Morquio A syndrome by applying noninvasive methods, such as electroglottography, acoustic analysis, and subglottal pressure measurement. Electroglottography (EGG) is a noninvasive technique for monitoring vibrations of the vocal folds by measuring the change in electrical impedance of the neck at the level of the vocal folds (Fabre 1957; Fourcin and Abberton 1971; Frokjaer-Jensen and Thorvaldsen 1968).

## Methods and patients

The study included 13 patients with Morquio A syndrome between 5.5 and 44 years old (divided into groups 1 and 2 by age), with a control group of 13 healthy individuals between 7.5 and 36 years old (groups 3 and 4, similarly divided by age).

Groups 1 and 3 included patients who were up to 18 years of age, while groups 2 and 4 consisted of those who were over 18 years old (Table 1). In group 1, all patients move with difficulty, except for patients 4, 5, and 6, who use a wheelchair. All patients in group 1 have severe clinical status. In group 2, there are two patients classified as moderate and three patients classified as mild. We call a phenotype severe if bone and joint changes are substantial, the stature very short, and the curvature of the spine and chest deformities are present. Inability to walk independently or at all, due to myelopathy, is present. We call a phenotype mild if the changes are less substantial, the short stature less evident, and the mobility is satisfactory. We call a phenotype moderate if the stature shortness is dominant and there are some limitations in mobility.

**Table 1:** Patient demographics – groups 1, 2, 3, and 4

Group no	ID	Gender	Current age	Cm/percentile	Kg/per centile	Mutation	Clinical status	Control group	Gender	Current age
Group 1	1	M	5.5	81.5 < 3c	10.5 < 3c	680delT/680delT	Severe	Control group 3	F	7.5
	2	M	8.5	86.7 < 3c	12.7 < 3c	G168R/ G168R	Severe		F	9
	3	F	9	112 < 3c	27.3 25-50c	G47R/R386C	Severe		M	9
	4	M	10.5	101 < 3c	17 < 3c	G155E/G155E	Severe wheelchair		M	10
	5	M	12.5	93 < 3c	19.2 < 3c	D183Y/D183Y	Severe wheelchair		M	10.5
	6	M	13.5	109 < 3c	22.5 < 3c	A231G/N407H/W520X	Severe wheelchair		F	11
	7	M	13.5	135.9 < 3c	37 3-10c	F227Sfs*92/121-9 T > G	Severe		M	11
	8	M	13.5	91.3 < 3c	17.5 < 3c	G168R/D233N	Severe		F	12
AVG age			10.8					AVG age		10.0
Median age			11.5					Median age		10.3
Group 2	9	F	18	108.8 < 3c	32.2 < 3c	G168R/ G168R	Moderate	Control group 4	F	20
	10	M	28	140.5 < 3c	52.2 3-10c	−/−	Mild		F	32
	11	F	31	91.8 < 3c	31.6 < 3c	G47R/G47R	Moderate		M	32
	12	F	33.5	157 = 3c	49 < 3c	R259Q/R94G	Mild		F	35
	13	M	44	–	–	−/−	Mild		M	36
AVG age			30.9					AVG age		31
Median age			31					Median age		32
AVG age 1.2.			18.5					AVG age 3.4.		18.1
Median age 1.2.			13.5					Median age 3.4.		11.0

Table 1. −/− Not found. Group 1 includes patients under 18 years of age with MPS IV A disease. Group 2 includes patients over 18 years of age with MPS IV A disease. Groups 3 and 4 include healthy patients under and over 18 years of age, respectively.

Both the patients and the control group took part in electrographic acoustic recordings and measurement of subglottal pressure. The recordings were collected at one point for each patient.

Permission to conduct the study (133/KBE/2014) was granted by the Bioethics Committee of the Children’s Memorial Health Institute in Warsaw. All participants gave informed, written consent prior to their participation, in a manner approved by the committee. Consent on behalf of the children enrolled was also obtained.

### Statistical analysis

Differences in the peak slope (PS), normalized amplitude quotient (NAQ), cepstral peak prominence (CPP), harmonics-to-noise ratio parameters (HNR) (described in “Acoustic analysis parameters” section) between Morquio A syndrome patients and healthy controls were calculated using independent samples T-tests. The differences between the jitter and the shimmer parameters were calculated using the U Mann-Whitney test, as the variances for these parameters differ despite the normal distribution (jitter *p* < 0.0001, F value 63.234, shimmer p < 0.0001, F value 16.262). The Anderson-Darling test was used to test normal distribution in each parameter. An F-test was calculated for each parameter to check if the variances were equal.

## Methods of voice quality evaluation

The study was conducted with the following Glottal Enterprises devices: the EG2-PCX2 electroglottograph and the PG-20E Subglottal Pressure Measurement System. The patients phonated with a close-to-natural volume and with a neutral fundamental frequency (F_0_)_._

### Electroglottography

Invented by Fabre (Fabre 1957) and further developed by Fourcin (Fourcin and Abberton 1971) and Frokjaer-Jensen, electroglottography (EEG) is a common method for providing noninvasive measurement of glottal activity. In the EEG measurement, a physiologically safe high-frequency, low-voltage, and low-intensity current passes between two electrodes mounted at the level of the thyroid cartilage. As the glottis opens and closes, impedance increases and decreases accordingly. By recording the pattern of vocal fold contact as the vocal folds vibrate, an EGG recording enables a thorough phoniatric examination and makes it possible to detect abnormalities in the patient’s voice.

Commercially available EEG devices are produced by Glottal Enterprises, Laryngograph Ltd., KayPENTAX and Voce Vista.

For EGG recordings, patients phonated the vowel /a/ three times for a sustained period with neutral volume.

### Acoustic analysis parameters

The microphone signal obtained through electroglottographic recordings was used for the acoustic analysis. The vowel /a/ was phonated three times for a sustained period with neutral volume. These recordings were used to assess vocal fold vibration and voice quality. The MATLAB (COVAREP toolkit) was also used for further analysis of PS, NAQ, CPP parameters (Degottex et al. 2014). The HNR, jitter, and shimmer parameters were calculated with Praat (Boersma 2001).

Six parameters (PS, NAQ, CPP, HNR, jitter, shimmer) were used to evaluate changes in voice quality in patients with Morquio A syndrome. The measurements were carried out for the vowel /a/ during sustained phonation.

**Peak slope (PS)** assists in distinguishing between breathy modal and tense voice. Furthermore, the peak slope algorithm can function as a standalone program independent of other algorithms (e.g., F_0_, inverse filtering). The method involves wavelet-based decomposition of the speech signal into octave bands and then fitting a regression line to the maximum amplitudes at different scales. The slope coefficient is used as the peak slope parameter (Degottex et al. 2014; Kane and Gobl 2011).

**Normalized amplitude quotient (NAQ)** has been used to effectively separate types of phonation (Alku et al. 2002). NAQ is a time-based parameter that measures sub-length of the glottal closing phase from two amplitude-domain measures. It is defined as the ratio of the AC flow amplitude to the negative peak amplitude of the flow derivative, normalized by the period length.

**Cepstral peak prominence (CPP**) is a parameter that enables the detection of early dysphonia. It is a very reliable voice analysis algorithm that measures the degree of harmonic structure within a voice signal. Being a measure of periodicity, it has been shown to correlate well with perceptions of breathiness. A cepstrum is a log power spectrum of a log power spectrum. The CPP measure is the difference in amplitude (in dB) between the peak and the corresponding value on the regression line that is directly below the peak (Hillenbrand and Houde 1996a, b; Maryn et al. 2009).

**Harmonics-to-noise ratio (HNR**) represents the degree of acoustic periodicity (Yumoto 1982; Hiraoka 1984). In Praat, it is expressed in dB. For example, if 99% of the energy of a signal is in the periodic part and 1% is noise, than the HNR is 10*log10(99/1) = 20 dB. During a sustained phonation of the vowel /a/, the value of this parameter in a healthy individual is 20 dB, whereas it falls below 20 dB in a person with a hoarse voice.

**Jitter local** is a measurement of vocal stability. It is calculated as an average absolute difference between consecutive periods, divided by the average period. The definition of pathology is any jitter local value ≥1.040% (Boersma 2001).

**Shimmer local** is also a measurement of vocal stability in terms of amplitude. It is calculated as an average absolute difference between the amplitudes of consecutive periods, divided by the average amplitude. The definition of pathology is any shimmer local value ≥3.81% (Boersma 2001).

### Subglottal pressure

Phonation occurs when the vocal folds are sufficiently close to each other. The air stream that passes between the folds creates a pressure drop in the glottis, which brings the vocal folds still closer together and causes the glottis to close. When expelled from the lungs, air then pushes the vocal folds open. This mechanism is repeated throughout the phonation process.

Subglottal pressure has been difficult to measure noninvasively. However, it has now become possible using a version of the intraoral pressure interpolation technique (Rothenberg 1973). Abnormalities in subglottal pressure measures can be indicative of impedance in the vocal tract, neuromuscular abnormalities of the chest wall, and pulmonary disease (Baken and Orlikoff 2000). For the measurement of subglottal pressure, patients repeatedly phonated the ‘pa-pa-pa’ syllables at the rate of 3–4 per second.

## Results

**Graph 1:**
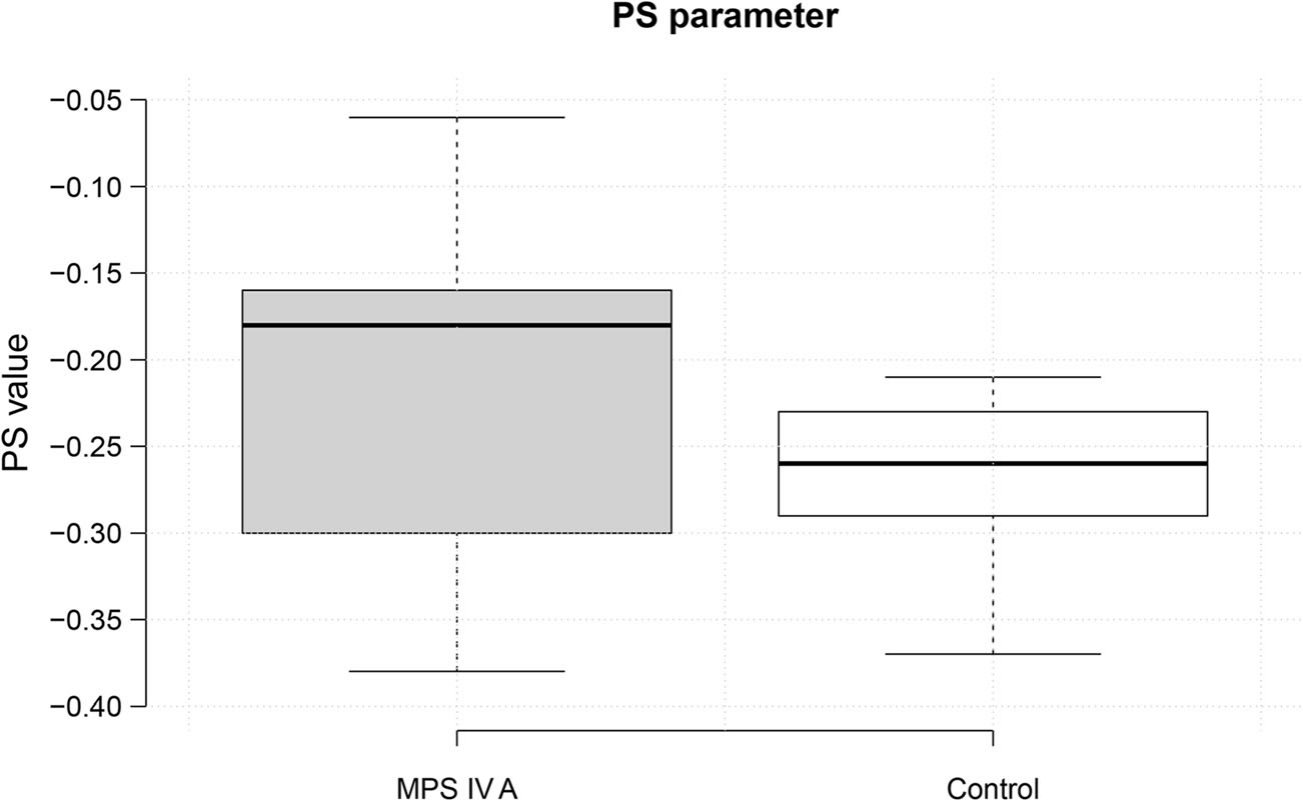
The values of the peak slope parameter. The graph on the left is for patients with MPS IV A, while the graph on the right is for the control group. The parameter values are on the Y axis. The median value (horizontal line) on the left-hand graph illustrates values characteristic of breathy voice in Morquio A syndrome. Higher values indicate a higher degree of breathiness. The median value on the graph for the control group indicates standard values for modal voice

**Graph 2:**
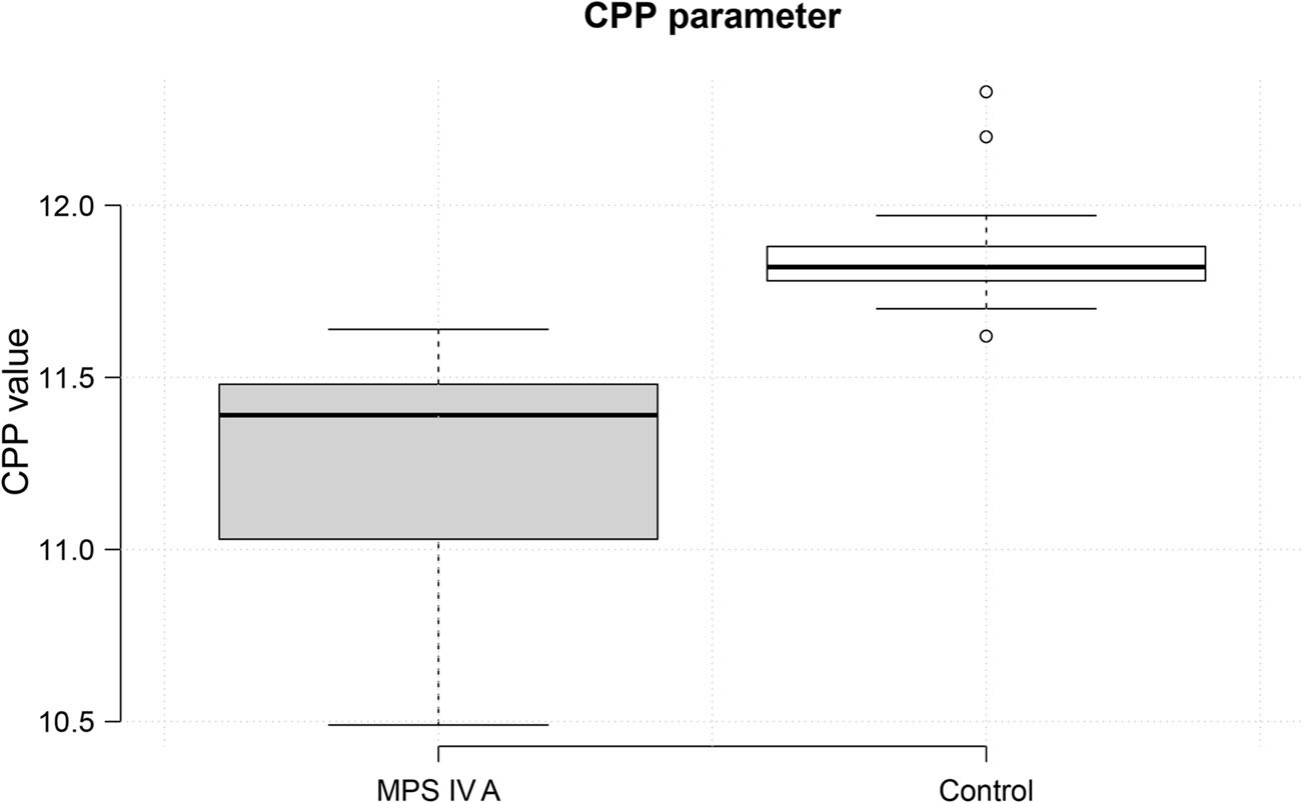
The values of the CPP parameter. The graph on the left is for patients with MPS IV A, while the graph on the right is for the control group. The parameter values are on the Y axis. The median value (horizontal line) on the left-hand graph illustrates values characteristic of dysphonic voice in Morquio A syndrome patients. The median value on the graph for the control group indicates standard values for modal voice

**Graph 3:**
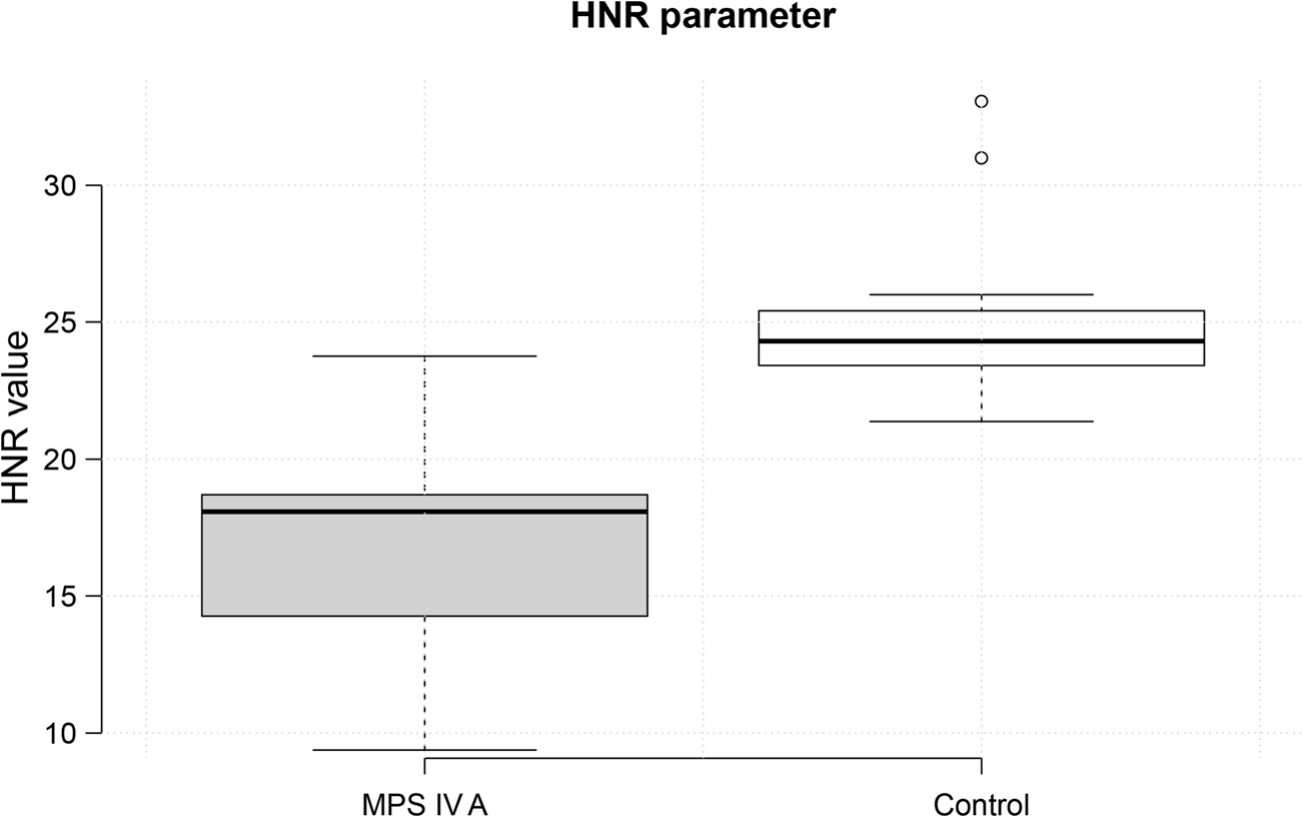
The values of the HNR parameter. The graph on the left is for patients with MPS IV A, while the graph on the right is for the control group. The parameter values are on the Y axis. The median value (horizontal line) on the left-hand graph illustrates values characteristic of hoarse voice in Morquio A syndrome patients. Values below 20 dB are characteristic for hoarse voice. The median value on the graph for the control group indicates standard values for modal voice

The results of the acoustic analysis for Morquio A syndrome patients and control groups are shown in Graphs 1, 2, and 3 and in Table 2.

**Table 2:** Mean values and standard deviation of peak slope, NAQ, CPP, HNR, jitter and shimmer parameters, and subglottal pressure values for all groups

Parameter/group	Group 1	Group 2	Group 3 control	Group 4 control	*p* value
PS	−0.16 ± 0.07	−0.28 ± 0.08	−0.23 ± 0.02	−0.33 ± 0.03	0.0394*
NAQ	0.1 ± 0.02	0.12 ± 0.03	0.15 ± 0.01	0.15 ± 0.01	0.0435*
CPP	11.31 ± 0.22	11.16 ± 0.47	11.82 ± 0.17	11.95 ± 0.22	<0.0001*
HNR	18.33 ± 2.97	13.27 ± 3.85	25.4 ± 3.24	24.89 ± 3.85	<0.0001*
Jitter local %	0.46 ± 0.19	1.29 ± 1.02	0.28 ± 0.10	0.31 ± 0.07	0.0025*
Shimmer local %	3.41 ± 1.35	5.64 ± 2.2	1.96 ± 0.07	1.62 ± 0.36	0.0003*
Cm H_2_O	5.21 ± 1.01	6.72 ± 2.25	7.54 ± 0.89	5.72 ± 0.84	0.1

The results are reported as mean ± standard deviation; The results of t-test are also reported

*Statistically significant

*PS*, peak slope; *NAQ*, normalized amplitude quotient, *CPP*, cepstral peak prominence; *HNR*, harmonics-to-noise ratio, *Cm H*_*2*_ *O* – subglottal pressure

Mean values of subglottal pressure necessary for phonation were within normal limits at 5.21 ± 1.01 and 6.72 ± 2.25 cm H_2_O for groups 1 and 2, and 7.54 ± 0.89 and 5.72 ± 0.84 cm H_2_O for groups 3 and 4, respectively.

The vocal fold frequencies (F_0_) were lower in boys and adult males with Morquio A syndrome (219 Hz and 101 Hz, respectively) than those in age-matched controls (253 Hz and 145 Hz, respectively). The values for affected patients of both age groups fell within the bottom part of the reference range (Baken and Orlikoff 2000).

The EGG signal showed a deviation from the modal voice waveform, which is characteristic for the presence of vocal nodules. The electroglottographic analysis indicated vocal nodules in Morquio A syndrome. Amplitude perturbation was found in seven patients from group 1 and two patients from group 2.

Six patients in group 1, and three patients in group 2 were found to have tense voice. The NAQ parameter was considerably lower among affected individuals (*t* = 2.064, *p* = 0.0435).

Breathy phonation was found in seven patients from group 1 and two patients from group 2. The PS parameter was considerably lower in affected individuals than among unaffected controls (*t* = 2.178, *p* =  0.0394).

All patients were diagnosed with dysphonia. The CPP parameter was significantly lower when compared to matched controls (*t* =  5.707, p  <  0.0001). Hoarse voice, measured by HNR, was found in seven patients in group 1 and in all patients in group 2 (measured by HNR). Statistically, this parameter was significantly lower in patients than in the control group (*t* = 5.995, *p* < 0.0001).

Instability of vocal fold vibrations was found in three patients from group 1 and four patients from group 2. The jitter parameter in Morquio A syndrome patients is higher than in their matched controls (U = 143.5, *p* = 0.0025). The shimmer parameter in Morquio A syndrome patients is higher than in their matched controls (U = 154.5, *p* = 0.0003).

The voice parameters for all subjects in the control groups demonstrated measured values consistent with normal modal voice.

## Discussion

Change in voice quality is characteristic of Morquio A syndrome, and is a consequence of infiltration and compromise of connective tissue. The aim of this study was to evaluate a new method of measuring subglottal pressure values and the functioning of the vocal folds in the context of this disease. The authors of this study (Szklanny et al. 2016) have previously demonstrated that an analysis of the incomplete glottal closure in Pompe disease is feasible. This method can also be used in other diseases. The method proposed in this study enables a noninvasive voice analysis. This is vital for Morquio A syndrome patients, where videofiberoscopy examination cannot be carried out due to atlantoaxial instability (Yeung et al. 2009).

In our analysis, mean values for subglottal pressure necessary for phonation were found to be normal across all study groups, at 5.21 ± 1.01 and 6.72 ± 2.25 cm H_2_O for groups 1 and 2, respectively, and 7.54 ± 0.89 and 5.72 ± 0.84 cm H_2_O for groups 3 and 4, respectively. Estimates of subglottal pressure for children aged 4 to 7 years at comparable intensities are between 5.2 and 9.72 cm H_2_O and decrease with age.

Children older than 13 years are expected to have subglottal pressure measurements between 3.9 and 8.04 cm H_2_O (Keilmann ABader 1995). One study showed that mean measures of subglottal pressure in the disease groups ranged from 8.72 cm H_2_O to 9.87 cm H_2_O (Weinrich et al. 2005). This suggests difficulties with articulation related to the function of vocal folds or to their structure in closed position.

The results of acoustic analysis demonstrated that individuals with Morquio A syndrome tended to have strained voice (the NAQ parameter) leading to incomplete glottal closure (the PS parameter). This finding was confirmed by the electroglottographic signal. The presence of vocal nodules was found in seven patients in group 1 and two patients in group 2. Vocal hyperfunctioning and laryngeal muscle tension increase the incidence of the development of vocal nodules.

Hyperfunctional phonation, accompanied by an increased tension in the laryngeal muscles, is the most likely cause of vocal nodules. Morrison et al. described four types of functional voice disorder (muscle tension dysphonia, MTD) (Morrison and Rammage 1993). Type 1 MTD, of relevance here, is characterized by incomplete glottal closure in the anterior and posterior glottis caused by excessive tension in the intrinsic laryngeal muscles and, most importantly, by incomplete relaxation of the posterior cricoarytenoid muscles. Incomplete glottal closure leads to localized tissue damage where the vocal folds are in full contact, usually at the junction of the anterior third of the vocal fold (Morrison and Rammage 1993; Dejonckere and Kob 2009).

In MPS IV A syndrome, increased vocal fold tension to achieve glottal closure and a strong irregularity of vocal folds vibration is observed (Nakarat et al. 2013). This leads to vocal nodules and is related to connective tissue elasticity.

This phenomenon affects other voice parameters (CPP, HNR). Dysphonia was found in all of the patients examined. The values of the CPP and HNR parameters were consistent with the etiology described above. Statistically, the mean value of the CPP parameter in Morquio A syndrome patients (11.25 ± 0.33 dB) was significantly lower than in the control group (11.86 ± 0.20 dB). The mean value of the HNR parameter in Morquio A syndrome patients (16.38 ± 4.12 dB) was significantly lower than in the control group (25.20 ± 3.34 dB). The pathologic threshold is 20 dB for HNR. Values below this are characteristic of hoarse voice.

Two patients had a measured jitter parameter outside the normal range (15% of all patients). The mean value of this parameter in Morquio A syndrome patients was 0.78% ± 0.74, while it was considerably lower in the control group (0.29% ± 0.09). Mean values fell within normal limits for both groups.

The mean value of the shimmer parameter for affected individuals was (4.27% ± 1.99). More than half (54%) of all patients had a shimmer parameter outside of normal limits, indicating pathology. By comparison, the mean value in the control group is 1.83% ± 0.49.

Previous studies have used acoustic analysis to evaluate voice alterations, articulations, and phonation patterns in patients with MPS I, II, and VI (Nakarat et al. 2013). In particular, Nakarat et al. analyzed parameters connected with voice, i.e., F_0_, jitter, shimmer, and glottal to noise excitation ratio (Nakarat et al. 2013). They found that values of the jitter parameter as shown in (Nakarat et al. 2013) exceed the norm in approximately 45% of MPS VI, 60% of MPS II, and 80% of MPS I patients. For the shimmer parameter, about 90% of MPS VI, 92% of MPS II, and 100% MPS I patients have values outside normal limits. The authors found that the fundamental frequency values (F_0_) are correct for both the MPS I, II, and VI patients and their matched controls.

These vocal abnormalities may be explained by the accumulation of glycosaminoglycans (GAGs) in the vocal tract, which leads to an unbalanced enlargement and coarsening of the structures of the vocal apparatus. In turn, this produces irregular vibration and variation in F_0_ (jitter) as well as changes in the amplitude of the sound signal between vibratory cycles (shimmer).

Unlike in MPS I, II, and VI, in which the accumulation of glycosaminoglycans leads to the reduced flexibility of the connective tissue, in Morquio A syndrome the connective tissue is structurally weakened rendering it unable to perform its function as supporting stroma tissue. For this reason, it is difficult to compare among acoustic analyses of these different diseases. The authors employed a set of diagnostic techniques (jitter, shimmer, glottal to noise ratio), which also affects the interpretation of the results. The analysis performed in this study provides a more comprehensive, quantitative understanding of the vocal changes observed in individuals with Morquio A syndrome.

## Conclusions

Alterations in voice quality are caused by disturbed connective tissue structure manifesting itself as severe limpness in Morquio A syndrome. This results in incomplete glottal closure, dysphonia, tense voice, hoarse voice, and the development of vocal nodules.

The application of an objective voice analysis allowed us to observe differences in vocal apparatus function between individuals with Morquio A syndrome and healthy controls. This offers an additional means to study the vocal apparatus over the course of the disease and to potentially measure the effects of enzyme replacement therapy.

## References

[CR1] Alku P, Bäckström T, Vilkman E (2002). Normalized amplitude quotient for parametrization of the glottal flow. J Acoustical Soc Am.

[CR2] Baken RJ, and Orlikoff RF (2000) Clinical measurement of speech and voice. Cengage Learning, Boston

[CR3] Boersma P (2001). Praat: doing phonetics by computer. Ear Hear.

[CR4] Degottex G, Kane J, Drugman T, Raitio T, Scherer S (2014) “OVAREP — A collaborative voice analysis repository for speech technologies”, 2014 I.E. International Conference on Acoustics, Speech and Signal Processing (ICASSP), Florence, pp 960–964. 10.1109/ICASSP.2014.6853739

[CR5] Dejonckere PH, Kob M (2009). Pathogenesis of vocal fold nodules: new insights from a modelling approach. Folia Phoniatrica et Logopaedica.

[CR6] Fabre P (1957). Un procédé électrique percutané d’inscription de l’accolement glottique au cours de la phonation: glottographie de haute fréquence. Bull Acad Natl Med.

[CR7] Fourcin AJ, Abberton E (1971). First applications of a new laryngograph. Med Biol Illustration.

[CR8] Frokjaer-Jensen B, Thorvaldsen P (1968). Construction of a Fabre glottograph. ARIPUC.

[CR9] Harmatz P, Mengel K, Giugliani R, Valayannopoulos V, Lin S, Parini R (2013). The Morquio a clinical assessment program: baseline results illustrating progressive, multisystemic clinical impairments in Morquio a subjects. Mol Genet Metab.

[CR10] Harmatz P, Mengel K, Giugliani R, Valayannopoulos V, Lin S, Parini R (2015). Longitudinal analysis of endurance and respiratory function from a natural history study of Morquio a syndrome. Mol Genet Metab.

[CR11] Hendriksz C, Harmatz P, Beck M, Jones S, Wood T, Lachman R (2013). Review of clinical presentation and diagnosis of mucopolysaccharidosis IVA. Mol Genet Metab.

[CR12] Hendriksz C, Lavery C, Coker M, Ucar S, Jain M, Bell L (2014). Burden of disease in patients with Morquio a syndrome: results from an international patient-reported outcomes survey. Orphanet J Rare Dis.

[CR13] Hillenbrand J, Houde RA (1996). Acoustic correlates of breathy vocal quality: dysphonic voices and continuous speech. J Speech Language Hearing Res.

[CR14] Hillenbrand J, Houde RA (1996). Acoustic correlates of breathy vocal quality dysphonic voices and continuous speech. J Speech Language Hearing Res.

[CR15] Hiraoka N (1984). Harmonic-intensity analysis of normal and hoarse voices. J Acoustical Soc Am.

[CR16] Kane J, Gobl C (2011) Identifying regions of non-modal phonation using features of the wavelet transform. INTERSPEECH, Beijing, p 177–180

[CR17] Keilmann A, Bader C (1995) Development of aerodynamic aspects in children’s voice. Int J Pediatr Otorhinolaryngol 31(2–3):183–190 10.1016/0165-5876(94)01089-g 7782176

[CR18] Maryn Y, Roy N, De Bodt M, Van Cauwenberge P, Corthals P (2009). Acoustic measurement of overall voice quality: a meta-analysis. J Acoustical Soc Am.

[CR19] Morrison MD, Rammage LA (1993). Muscle misuse voice disorders: description and classification. Acta Otolaryngol.

[CR20] Nakarat T, Läßig A, Lampe C, Keilmann A (2013). Alterations in speech and voice in patients with mucopolysaccharidoses. Logopedics Phoniatrics Vocology.

[CR21] Nashed A, Al-Saleh S, Gibbons J, MacLusky I, MacFarlane J, Riekstins A, Clarke J, Narang I (2009) “Sleep-related breathing in children with mucopolysaccharidosis”. J Inherit Metab Dis 32(4):544–550. 10.1007/s10545-009-1170-4 10.1007/s10545-009-1170-4 19562504

[CR22] Pelley CJ, Kwo J, Hess DR (2007). Tracheomalacia in an adult with respiratory failure and Morquio syndrome. Respir Care.

[CR23] Rothenberg M (1973). A new inverse-filtering technique for deriving the glottal air flow waveform during voicing. J Acoustical Soc Am.

[CR24] Szklanny K, Gubrynowicz R, Iwanicka-Pronicka K, Tylki-Szymańska A (2016) Analysis of voice quality in patients with late-onset Pompe disease. Orphanet J Rare Dis 11(1). 10.1186/s13023-016-0480-5 10.1186/s13023-016-0480-5 PMC4946183 27417441

[CR25] Walker P (2003). Upper airways abnormalities and tracheal problems in Morquio's disease. Thorax.

[CR26] Weinrich B, Salz B, Hughes M (2005). Aerodynamic measurements: normative data for children ages 6:0 to 10:11 years. J Voice.

[CR27] Yeung A, Cowan M, Horn B, Rosbe K (2009). Airway management in children with Mucopolysaccharidoses. Arch Otolaryngology–Head Neck Surg.

[CR28] Yumoto E (1982). Harmonics-to-noise ratio as an index of the degree of hoarseness. J Acoustical Soc Am.

